# Mechanobiology of portal hypertension

**DOI:** 10.1016/j.jhepr.2023.100869

**Published:** 2023-08-02

**Authors:** Eric Felli, Sonia Selicean, Sergi Guixé-Muntet, Cong Wang, Jaume Bosch, Annalisa Berzigotti, Jordi Gracia-Sancho

**Affiliations:** 1 Department of Visceral Surgery and Medicine, Inselspital, Bern University Hospital, University of Bern, Switzerland; 2 Department for BioMedical Research, Visceral Surgery and Medicine, University of Bern, Switzerland; 3 Liver Vascular Biology Research Group, IDIBAPS Biomedical Research Institute, CIBEREHD, Spain

**Keywords:** Liver cirrhosis, liver sinusoidal endothelial cells, LSEC, liver fibrosis, hepatic stellate cells, HSC

## Abstract

The interplay between mechanical stimuli and cellular mechanobiology orchestrates the physiology of tissues and organs in a dynamic balance characterized by constant remodelling and adaptative processes. Environmental mechanical properties can be interpreted as a complex set of information and instructions that cells read continuously, and to which they respond. In cirrhosis, chronic inflammation and injury drive liver cells dysfunction, leading to excessive extracellular matrix deposition, sinusoidal pseudocapillarization, vascular occlusion and parenchymal extinction. These pathological events result in marked remodelling of the liver microarchitecture, which is cause and result of abnormal environmental mechanical forces, triggering and sustaining the long-standing and progressive process of liver fibrosis. Multiple mechanical forces such as strain, shear stress, and hydrostatic pressure can converge at different stages of the disease until reaching a point of no return where the fibrosis is considered non-reversible. Thereafter, reciprocal communication between cells and their niches becomes the driving force for disease progression. Accumulating evidence supports the idea that, rather than being a passive consequence of fibrosis and portal hypertension (PH), mechanical force-mediated pathways could themselves represent strategic targets for novel therapeutic approaches. In this manuscript, we aim to provide a comprehensive review of the mechanobiology of PH, by furnishing an introduction on the most important mechanisms, integrating these concepts into a discussion on the pathogenesis of PH, and exploring potential therapeutic strategies.

**Key points**

• Accumulating data highlight mechanobiology as a crucial initiator and modulator of physiological and pathological cell behaviour.
• Liver cells are exposed to a variety of mechanical forces under homeostatic conditions, and these become aberrant during the development of cirrhosis.
• Chronic altered mechanical stimulation leads to dysfunctional liver cells and contributes to the maintenance and progression of portal hypertension.
• Targeting mechanosensing and mechanotransduction pathways may aid in reversing phenotypical alterations of cirrhotic liver cells.
• A deeper understanding of the ways in which altered mechanobiological cues modulate the progression and maintenance of chronic liver disease may help in the design of new therapeutic strategies for portal hypertension.

## Introduction on mechanobiology

Mechanical forces exerted by cells underlay a dynamic balance that orchestrates the physiology of all living organisms. In fact, tissues and organs are characterized by complex architectures that result from continuous remodelling modulated by cell mechanosensing. The balance is maintained internally via cellular tension-generated forces from the cytoskeleton, and externally by mechanical stimuli from the environment. From this perspective, environmental mechanical properties can be interpreted as a complex set of information and instructions that cells read continuously, and to which they adapt. Mechanical forces can be considered an upstream driver of a cell’s phenotype in the field of mechanobiology, but underlying pathological events represent the primary factor that disrupts the complex balance of mechanosensing.^1^^,^^2^ This is the case in liver fibrosis, where mechanical forces gradually take the lead in the progression of the disease, sustaining and driving liver cell dysfunction. Indeed, in chronic liver diseases (CLDs) of different aetiologies, chronic inflammation and injury initiate progressive cell dysfunction, altering liver microarchitecture, from which cells receive pathological stimuli in a long-standing and progressive process.^3^ At this point, multiple mechanical alterations converge at different stages of the disease in a winch-like loop, until reaching a point of no return, where the fibrosis is considered non-reversible. Following this, the reciprocal communication between cells and their niche can be altered in many ways – *e.g.* by tensile stress, hydrodynamic pressure, and shear stress, with resulting cell stretch and/or compression – becoming the driving force for disease progression. These mechanical forces, at the cellular and molecular level, have recently been recognized as a driver of liver pathology,[4], [5], [6], [7] and thus a potential target for novel therapeutic approaches.

### Mechanosensing and mechanotransduction

**Fig. 1:**
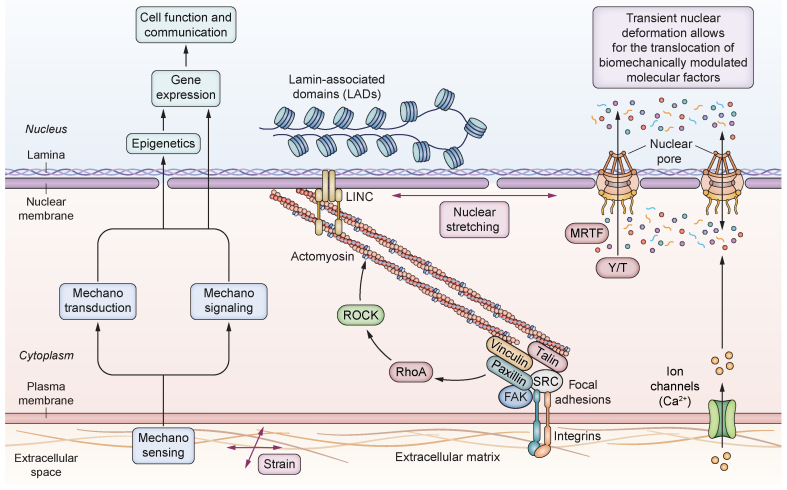
Strain-related elements of the mechanotransduction cascade. A stiff underlying matrix leads to the formation of focal adhesions, which activate downstream pathways leading to increased contraction of the cytoskeleton. This in turn acts on the nuclear membrane, changing the conformation of nuclear pores and/or leading to epigenetic changes. FAK, focal adhesion kinase; LINC, linker of nucleoskeleton and cytoskeleton; MRTF, myocardin-related transcription factor; ROCK, Rho-associated protein kinase; Y/T, Yes-associated protein 1/WW-domain-containing transcription regulator 1.

The mechanosensing machinery of cells spans from the extracellular environment all the way to the interior of the nucleus. Externally, forces are transmitted from the extracellular space to the cell through transmembrane proteins, as well as directly from the blood flow. Internally, due to repetitive contraction and relaxation of the actin filaments of the cytoskeleton, cells sense the stiffness or pressure of their environment, generating a tension proportional to it. In the case of extracellular matrix (ECM)-cell attachment, this intracellular tension pulls on the ECM-bound integrins, which then organize into focal adhesions (FAs) along with adaptor proteins to reinforce the ECM-cytoskeleton link. The cytoskeleton-generated tension is then transmitted to the nucleus by the LINC (linker of nucleoskeleton and cytoskeleton) complex. This can be bound either to the nuclear pore complex or to the lamins, which are connected to the lamin-associated domains (LADs) on chromatin.^8^^,^^9^ Consequently, internal tension generated by the cytoskeleton during mechanosensing and mechanotransduction deforms the nucleus proportionally to the force generated by or applied to the cell, altering the permeability of its pores and the traffic of biomechanically modulated molecular factors, and ultimately influencing gene expression by directly modifying the chromatin structure and the permeability of the cell and nuclear membranes (Fig. 1).

### ECM-nucleus axis

#### Mechanosensing at the plasma membrane

Transmembrane proteins, such as integrins, play a key role in mechanobiology, allowing cells to sense the environment. Integrin biology is highly complex, and their involvement in disease is wide-ranging.^10^ Upon stiffness sensing, integrins bind to talin, causing its unfolding, revealing cryptic binding sites, and recruiting vinculin^11^ and other cytoskeleton linker proteins. This then leads to the activation of downstream signalling proteins. Moreover, integrins are also involved in transforming growth factor-β (TGFβ) signalling, allowing its release from the ECM-bound latency associated peptide, thanks to stiffness-mediated conformational changes.^12^ Besides integrins, ion channels (*e.g.*, Ca^2+^) play an important role in plasma membrane mechanosensing.^13^ Any force applied on the cell membrane alters the conformation of ion channels, allowing for passive transport of ions with marked consequences on cell osmolarity, and protein and chromatin stability. TRPV4 (transient receptor potential cation channel subfamily V member 4) and Piezo1^14^ are ion channels that respond to a wide variety of mechanical stimuli, such as shear stress,^15^ membrane stretch,^16^ or pressure,^17^ and may also be involved in vascular or immune aspects of advanced CLD, as they play such roles in other systems.^18^^,^^19^ Furthermore, caveolae,^13^ another mechanosensitive plasma membrane-associated component, may be involved in maintaining endothelial homeostasis, as extensively reviewed elsewhere.^20^

#### Regulation of the cytoskeleton

Downstream of integrins and FAs, several proteins guarantee transduction of mechanical forces generated by the cytoskeleton. Rho-GTPases play crucial roles in actin nucleation and elongation and in actomyosin contractility. Rho-associated coiled-coil-containing kinase (ROCK), an important RhoA effector, is involved in myosin light chain phosphorylation, stimulating the interaction between myosin and actin. Moreover, it also inhibits actin depolymerization. The resultant turnover contributes to the dynamic remodelling of the cytoskeleton, supporting an increase in tension, which is responsible for downstream transmission towards the nucleus.^21^

#### Nuclear alteration

Cell contractility (*i.e*., during migration) imposes a transient compressive force through lateral and ventral actin fibres anchored to the outer nuclear membrane via the LINC complex. However, on flat, rigid substrates, actin fibre alignment produces stretching, compression, and indentation of the nucleus,^22^ which can lead to nuclear envelope damage.^23^ Nuclear compression and cytoskeletal tension are directly proportional to the rigidity of the ECM. Disruption of the actin cytoskeleton reduces compression on the nucleus, restoring cell functionality in high stiffness.^24^ Nuclear deformation can modulate gene expression mainly by altering chromatin rheology and nuclear membrane permeability. Chromatin is connected to the nuclear envelope, whose compression and stretching alters its conformation via LADs, as comprehensively reviewed elsewhere.^1^ Moreover, nuclear deformation controls chromatin localization, methylation, and acetylation,^25^^,^^26^ playing a key epigenetic role in gene transcription.^27^ When cells spread out on stiff substrates, stretching of the nuclear membrane induces dilation of the nuclear pore complex, which adopts a more open conformation, allowing for translocation of transcription factors or co-factors.^8^ Yes-associated protein 1/WW-domain-containing transcription regulator 1 (YAP/TAZ)^28^ are transcription co-factors whose effects in development and carcinogenesis have been widely described.^29^ Interestingly, recent studies have demonstrated that nuclear translocation of YAP/TAZ is dependent on the substrate stiffness in a non-linear manner and that they also play prominent roles as profibrotic factors.^30^^,^^31^ Similarly, subcellular localization of myocardin-related transcription factor-A (MRTF-A) is regulated by its association with cytoplasmic actin G, which, upon Rho signalling and polymerization into filamentous actin, releases MRTF-A that translocates to the nucleus and activates SRF (serum response factor).^32^ Zyxin, which is part of the mechanosensing FA complex, also translocates to the nucleus upon cell stretch and regulates genes related to apoptosis, proliferation, and inflammation.^33^

**Fig. 2:**
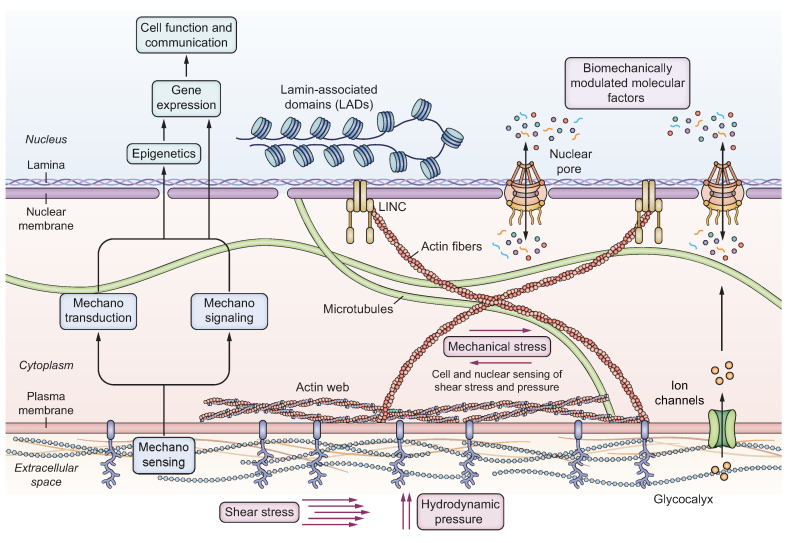
Shear stress and hydrodynamic pressure-related elements of the mechanotransduction cascade in endothelial cells. Blood flow stimulates endothelial cells through hydrodynamic pressure and shear stress, acting directly on the glycocalyx of the cell membranes, indirectly massaging the nucleus via the cytoskeleton network in a cyclic manner. LINC, linker of nucleoskeleton and cytoskeleton.

In endothelial cells, mechanical forces other than tensile stress play a key role in cellular homeostasis. Hydrodynamic pressure and shear stress resulting from the action of blood flow on cell membranes are transmitted by the actin cortex and the glycocalyx through the cytoskeletal network up to the nucleus (Fig. 2). These mechanical forces modulate the expression and localization of YAP and TAZ, which act in the nucleus as activators of TEAD, a family of biomechanically modulated molecular factors, contributing to the maintenance of cellular homeostasis.^34^ The vector of shear stress is tangential to endothelial cells, and its magnitude and direction are key regulators of gene expression. Flow transmits mechanical force to the nucleus via intracellular tension from the membrane modulating vascular endothelial-cadherin, PECAM1 (platelet and endothelium cell adhesion molecule 1), and VEGFR2 (vascular endothelial growth factor receptor). A cell’s adaption to various changes in blood flow, pressure, and turbulence involves the cytoskeleton-nuclear axis, as well as the biochemical modulation of molecular factors,^35^ and to a certain unknown extent their translocation due to nuclear deformation, as described below.

## Mechanobiology in portal hypertension

**Fig. 3:**
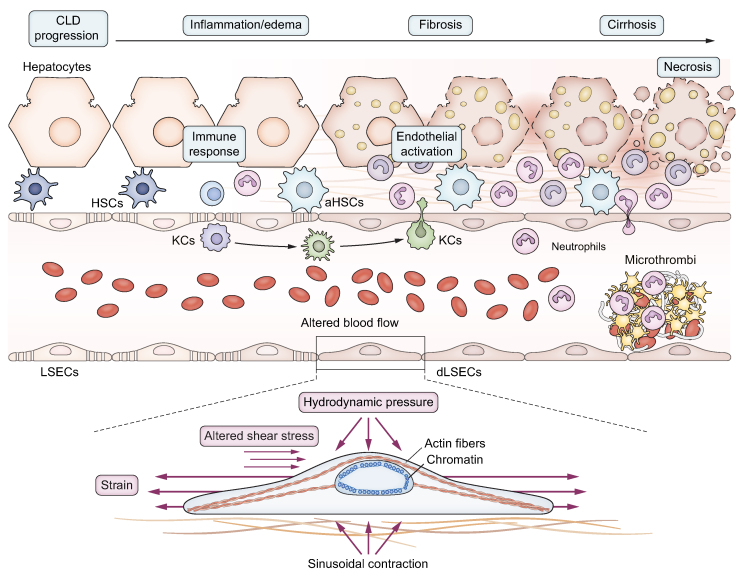
Mechanobiology in chronic liver disease and portal hypertension. During the progression of liver disease, several alterations with mechanical consequences take place: excessive extracellular matrix secretion and deposition (fibrosis), altered haemodynamics, sinusoidal hypercontraction, microthrombi, and interstitial oedema. Increase in hepatocyte size by ballooning and steatosis, and cholestasis could be additional features that may contribute to altered mechanosensing. These alterations produce different mechanical forces on the surrounding cells: increased stiffness sensing, distorted shear stress and pathological hydrodynamic pressure. Upon encountering these forces, cells are stretched and/or compressed, stimulating a chronic and pathological de-differentiation. (a)HSCs, (activated) hepatic stellate cells; KCs, Kupffer cells; (d)LSECs, (de-differentiated) liver sinusoidal endothelial cells.

Portal hypertension (PH) represents the most common and detrimental non-neoplastic complication of CLD and is diagnosed when the pressure gradient between the portal vein and the inferior vena cava (measured clinically in cirrhosis by the hepatic venous pressure gradient, or HVPG) exceeds 5 mmHg. It is considered clinically significant when the HVPG is ≥10 mmHg, as going beyond this threshold is associated with important clinical complications like variceal bleeding, ascites, infections, hepatorenal syndrome, and hepatic encephalopathy.^36^ The pathophysiology of PH starts with the de-differentiation of liver cells due to continued injury, development of hepatic microcirculatory dysfunction, and elevation of intrahepatic vascular resistance (IHVR). IHVR has two components: a structural one, derived from a long-standing fibrogenic process, characterized by distorted microcirculation due to altered microarchitecture, vascular occlusion, parenchymal extinction, and regenerative nodules,^37^ and a functional component, characterized by endothelial dysfunction and hepatic stellate cells (HSCs) hypercontraction^38^ leading to a dynamic increase in vascular resistance to blood flow. In this context, the activation of HSCs and liver sinusoidal endothelial cells (LSECs) dysfunction generate an altered cross-talk that further aggravates microvascular dysfunction. Several factors produced by LSECs, such as endothelin-1^39^ or nitric oxide (NO),^40^ can modulate HSCs activation and increase contractility and ECM production or, conversely, promote HSCs’ return to a quiescent phenotype.[41], [42], [43] Moreover, paracrine NO signalling modulates mechanobiology-related processes, such as the formation of FAs and migration of HSCs.^44^^,^^45^ The progressive intrahepatic accumulation of ECM, together with increased vascular tone, modifies the sinusoidal milieu, leading to changes in the mechanical properties of the liver and, consequently, to the adaptation of liver cells via mechanotransduction (Fig. 3). Although PH is considered partially reversible,^46^ during the progression of CLD, changes in the local environment may reach a point of no return where the disease is considered irreversible, which is thought to occur when liver scarring is dense, extensively cross-linked and almost acellular with a concomitant loss of parenchymal cell mass. Indeed, altered mechanosignalling may be the factor that drives progression of CLD to this non-reversible stage. This underlines the importance of studying mechanobiology to determine the point of no return in cirrhosis and to identify molecular targets that could guide novel therapeutic strategies.

### Mechanical cues influencing portal hypertension

#### Shear stress

Shear stress is defined as the tangential force resulting from the friction generated by blood flowing over the endothelial surface, whose magnitude depends on the blood flow and the area of the sinusoids. In the liver, functional vascular integrity ultimately depends on LSEC and HSC homeostasis. Shear stress is a constant stimulus that is crucial for the function of LSECs, which under physiological conditions release vasodilatory agents such as NO in response to blood flow in order to maintain a physiological sinusoidal pressure. In PH, LSECs dysfunction is characterized by the loss of this property, as well as changes in the release of paracrine/angiocrine factors,^47^ impairing hepatic sinusoid cell cross-talk, activating mechanosensing-related molecular pathways,^48^ and changing the membrane-related proteome profile.^49^ LSECs dysfunction may be the upstream event responsible for the structural and functional components of IHVR,^36^ playing a major role together with cytokines release in driving HSCs activation. Kruppel-like factor 2 (KLF2) is a transcription factor that sustains LSECs’ protective phenotype^50^ by regulating the release of NO and vasoconstrictors like endothelin-1. KLF2 expression rises with the magnitude of shear stress and is an early indicator of microcirculatory dysfunction in CLD.^51^ However, its role is still discussed due to the presence of two KLF2 transcripts generated by alternative splicing, that prevent it from being a direct therapeutic target for CLD.^52^ Unfortunately, the role of epigenetic changes in LSECs stressed by PH is still not clear; understanding how these changes affect the promoter of KLF2 at CpG islands would uncover a key aspect of the pathophysiology of CLD. In addition, decreased bioavailability of NO is sustained by the increased amount of reactive oxygen species (which are responsible for NO scavenging rate), by a deficit in tetrahydrobiopterin (a chaperone required for endothelial NO synthase [eNOS] coupling), and by an increased synthesis of asymmetric dimethyl-arginine (an endogenous eNOS inactivator).^53^ Finally, together with KLF2 expression, LSECs autophagy plays an important role in response to shear stress. A recent work from our group highlighted the protective role of a statin (simvastatin) in maintaining the cross-talk between autophagy and KLF2 expression via its inhibitory effect on Rac1.^54^

#### Hydrodynamic pressure

Sinusoidal stretch and pressure are also crucial in modulating cell phenotype, as suggested by data obtained in models of chronic liver congestion.^55^ Moreover, once it progresses, liver disease of any aetiology will encompass increased fluid pressure and abnormal cell stretch due to disturbed vascular flow, cholestasis, and interstitial oedema. Theoretically, once fibrosis develops, the tissue becomes less compliant and cell stretch will become less prominent, while at the same time, pressure within the sinusoids will increase and will be transmitted to the adjacent cells.

In other contexts, both forces have been shown to influence several processes in endothelial cells, as reviewed elsewhere.^56^ Comparatively few studies have so far investigated the effects of sinusoidal stretch and pressure during CLD development. Recently, our group has demonstrated the relative contribution of pathologic pressure to LSEC dysfunction in CLD and has described a pressure-sensitive transcription factor, CBX7 (chromobox homolog 7), modulated by miR-181a-5p. Moreover, expression of CBX7 significantly correlated with HVPG, while its downstream secreted proteins (ECAD and SPINK1) represent accurate biomarkers for assessing the presence of PH and CSPH.^57^ In congestive liver disease, upon endothelial cell stretch, a Piezo1-Notch1 receptor-Hes/Hey pathway has been described, which results in microthrombosis and increased hydrodynamic pressure, ultimately forming a feedback loop that aggravates disease progression.^16^ Thrombosis of small vessels may also play a role in liver diseases of other aetiologies,^37^ a hypothesis supported by the favourable effect of anticoagulant treatment in cirrhosis.^58^^,^^59^ Whether mechanical stimuli also play a role in the occurrence of microthrombosis outside of congestive liver disease is not yet established. Moreover, the swelling and increased size of lipid-laden hepatocytes have also been proposed to act as an external mechanical stimulus by encroaching upon the sinusoidal space and decreasing its lumen size, thus disrupting microvascular homeostasis early in the development of NAFLD (non-alcoholic fatty liver disease), and contributing to the development of PH, as reviewed elsewhere.[60], [61], [62]

#### Matrix stiffness

A characteristic of CLD is a progressive increase in liver stiffness due to excessive matrix deposition. A large amount of evidence in the clinical setting has shown that liver stiffness in patients with compensated CLD is a potent independent predictor of the presence of PH^63^ and of the development of first clinical decompensation.^64^ Interestingly, even in patients who developed a first decompensating event (in whom cirrhosis and PH are always present), a higher liver stiffness predicted recurrent decompensation and liver-related mortality.^65^ In a carbon tetrachloride (CCl_4_) model of murine cirrhosis, it was found that myofibroblastic differentiation preceded excessive ECM deposition and was related to increased stiffness due to LOX (lysyl oxidase)-mediated cross-linking of already present collagen fibres.^66^ Recent studies assessed the effects of increased matrix stiffness on the phenotype of cells isolated from healthy livers. Both hepatocytes and HSCs grown in high stiffness conditions (mimicking the stiffness of cirrhotic livers) undergo morphological and functional changes, adopting a more dedifferentiated and proliferative phenotype.[67], [68], [69] Hepatocytes diminish their synthetic and detoxifying functions,[67], [68], [69], [70] changes partially related to Rho/ROCK-modulated downregulation of HNF4a (hepatocyte nuclear factor-4α) at the nuclear level.^68^^,^^69^ In HSCs, markers of activation are increased,^67^^,^^70^^,^^71^ while peroxisome proliferator-activated receptor-γ (PPARγ) is decreased,^70^ and the activity of matrix metalloproteinase 9 and TIMP1 (TIMP metalloproteinase inhibitor 1) is altered, perpetuating the fibrotic process.^72^

Recently, our group demonstrated the effect of substrate stiffness on the phenotype of LSECs.^67^ When cultured on polyacrylamide (PAA) gels with high stiffness (30 kPa), healthy rat LSECs displayed a reduced number of fenestrations, increased laminin B1 and eNOS expression, and decreased NO synthesis, consistent with features of *in vivo c*apillarization. Altogether, this evidence suggests that, in the fibrotic liver, hepatic cells would be constantly activated due to high ECM stiffness, contributing to disease progression. However, the mechanisms of stiffness sensing in the liver remain largely unknown. In the liver, the previously described integrin-talin-cytoskeleton-nucleus pathway may also be crucial for stiffness sensing. Indeed, an increase in different subtypes of integrins has been described in CLD;^73^ however, integrin-mediated cytoskeletal mechanotransduction is not well understood in the different liver cell types. Although nuclear deformation is a direct consequence of increased matrix stiffness, deformation of the nucleus (and its downstream effects on gene expression) could occur. Indeed, nuclear localization of the transcriptional co-factor YAP, chromatin modifications, and altered cellular response to underlying matrix stiffness due to nuclear deformation are observed in lipid-loaded hepatocytes.^74^^,^^75^ Whether this mechanism might be part of the complex pathogenesis of hepatocellular carcinoma (HCC) in non-cirrhotic NAFLD livers remains to be ascertained. The mechano-responsive transcriptional co-factor YAP also modulates the phenotype of HSCs in response to increased stiffness via its cytoskeletal-dependent translocation to the nucleus, contributing to a profibrotic programme.^31^^,^^76^ *In vivo*, YAP and its target genes *Ctgf* and *Ankrd1* may be induced very early, after only one administration of CCl_4_, a timepoint when necro-inflammation is the only significant change that may increase tissue stiffness. This suggests the possible involvement of mechanoresponsive mechanisms in early disease stages, alongside their known role in later disease stages, which are characterized by a vicious cycle wherein increasing fibrosis maintains the nuclear localization of YAP that further promotes fibrosis.^30^ As a proof-of concept, YAP inhibition with verteporfin (visudyne) reduces fibrogenesis in CCl_4_ and bile duct-ligated murine models,^30^^,^^76^ therefore validating its relevance in the progression of the disease. In LSECs, the role of YAP in stiffness sensing has not yet been characterized, although it is likely to be relevant, as demonstrated by studies in other vascular beds. In pulmonary hypertension, increased matrix stiffness induces a YAP/TAZ-controlled metabolic switch towards increased glycolysis and glutaminolysis, thus supporting endothelial cell hyper-proliferation, endothelial-to-mesenchymal transition, and vascular remodelling.^77^ Moreover, a YAP/TAZ-miR130/301 circuit has been described, which both responds to increased matrix stiffness and promotes further ECM remodelling via the PPARy-APOE-LRP8-LOX pathway, thus highlighting the modified ECM as both cause and consequence of vascular lung diseases.^78^ Aside from YAP, the transcriptional co-factor MRTF-A has been shown to progressively increase in parallel with the rise in portal pressure in a CCl_4_ rat cirrhosis model.^79^ However, this study only investigated whole liver homogenates, which leaves open the question as to the specific function of MRTF-A in endothelial cells and other individual cell types. During activation of HSCs, MRTF-A and myocardin enhance cells’ contractility, motility, and proliferation, and impair SRF, Smad2/3 and Erk1/2-mediated fibrogenic signalling, resulting in a decrease in collagen expression.^80^ Alternatively, p300 is another transcriptional regulator involved in HSCs activation. Indeed, high matrix stiffness promotes AKT-mediated phosphorylation of p300 acetyltransferase, a post-translational modification that confers p300 with higher stability and mediates its nuclear translocation, with the subsequent activation of HSCs and upregulation of αSMA (α-smooth muscle actin) and CTGF (connective tissue growth factor).^81^ In TGFβ1-stimulated fibroblasts, p300 acts as a shuttle for nuclear translocation of SMAD2/3, and TAZ (but not YAP),^82^ a mechanism which might also be involved in stiffness sensing. Aside from altering nuclear transport, mechanical forces may also induce changes in chromatin conformation and other epigenetic mechanisms.^25^^,^^26^^,^^83^ In TGFβ1-stimulated fibroblasts, p300 drives expression of target genes by histone acetylation, while *in vitro* activation of HSCs causes MeCP2-mediated transcriptional repression of PPARγ.^84^^,^^85^ Whether these modifications are related to stiffness sensing, as described in other contexts, has not been determined. Lastly, the relationship between matrix stiffness and inflammation is worth noting. The capacity of neutrophils to transmigrate across the endothelium has been shown to increase proportionally with substrate stiffness in TNFα-activated endothelial cells and human umbilical vein endothelial cells grown on a stiff substrate have a stronger pro-inflammatory response to LPS stimulation, as evidenced by upregulation of interleukin-8, ICAM-1 (also known as CD54) and VCAM-1 (vascular cell adhesion molecule 1).^86^^,^^87^

Matrix stiffness also has an impact on intercellular cross-talk in the liver. Indeed, improvement of the phenotype of cirrhotic LSECs and HSCs in response to low matrix stiffness can improve the phenotype of other HSCs and LSECs, respectively, in a paracrine manner.^67^ Moreover, culture of LSECs on a high stiffness substrate can drive HSCs activation and ECM remodelling through the DDR2-JAK2/PI3K/AKT-myocardin signalling pathway, creating a profibrogenic feedback loop involving sinusoidal phenotype and ECM stiffness.^88^

#### Reversibility of the effects of mechanosensing

Importantly, the mechanosensing mechanism and its effects seem to be reversible. Freshly isolated HSCs, LSECs and hepatocytes from cirrhotic animals displayed an improvement in their phenotype, function, and paracrine communication, and showed an altered response to a drug with antifibrotic effects when plated on soft matrices or treated with cytoskeleton disruptors.^67^ Moreover, culture-activated HSCs grown on progressively softening matrices exhibited a reduction in spreading, nuclear YAP/TAZ and actin organization; however, they displayed an intermediate phenotype with reduced GFAP and rapid reactivation upon re-stiffening of the substrate.^89^ Indeed, YAP/TAZ are related to mechanical memory in mesenchymal cells and may play a role in the early reactivation of HSCs^90^ along with substrate-modulated epigenetic changes.^25^^,^^90^ This data suggests that modulating molecular pathways involved in stiffness sensing could represent novel therapeutic strategies for CLD regression.

Finally, the interplay between increased tissue stiffness and HCC, as a significant complication in patients with PH, also merits attention. In the clinical setting, a higher liver stiffness as measured by transient elastography is associated with increased risk of HCC development in patients with HCV^91^^,^^92^ and there is a positive correlation between ECM stiffness of HCC tissues and integrin β1 expression.^93^ *In vivo*, HCC growth is accelerated in fibrotic, stiff livers compared to healthy ones.^94^ *In vitro*, HCC cells respond to increased matrix stiffness through enhanced proliferation, a switch towards a mesenchymal phenotype, and inhibition of apoptosis.^95^^,^^96^ Possible mechanisms of carcinogenesis mediated by mechanosensing in the cirrhotic liver are downregulation of proteasomal degradation of YAP,^97^ mechanically induced chromatin modifications and genomic instability, which have been demonstrated in other tissues.^7^

## Opportunities to therapeutically target mechanobiological changes in liver fibrosis and portal hypertension

Generally, there are two major approaches when considering modulation of mechanosensitive pathways: firstly, targeting the mechanical input itself, by attenuating matrix stiffness (*e.g*., targeting fibrosis) or normalizing blood flow (*e.g.*, using vasoactive substances), and secondly, inhibiting the cellular response downstream of mechanical stimulation. Several methods aimed at reducing tissue stiffness have been tried in CLD; however, the most clinically advanced efforts have shown little success so far. Simtuzumab (lysyl oxidase like 2 antagonist), which targets ECM cross-linking, had no effect in NASH-, HCV/HIV-, or primary sclerosing cholangitis-related CLD due to redundant mechanisms contributing to tissue stiffening in more advanced stages of the disease.[98], [99], [100] Similarly, inhibition of galectin-3 (a pro-inflammatory and profibrotic factor) has shown basically no effect in NASH-related CLD.^101^ Several other approaches targeting tissue stiffness are being tested in the preclinical setting and may hold promise, such as inhibition of collagen I, ECM cross-linking or DDR1, as reviewed elsewhere,^102^ although these therapies are likely to be more effective at inhibiting disease progression than reversing more advanced disease. An alternative to targeting the mechanical properties of the ECM may be to modulate how cells respond to these altered mechanical features of the environment. In this sense, several potential druggable targets exist. Integrins are the first ones to sense and transmit mechanical stimuli from the ECM to the cell and thus their inhibition is an attractive therapeutic option. One integrin inhibitor (PLN-1474, an integrin ανβ1 inhibitor) has been tested in phase I clinical trials in the context of liver fibrosis. Preclinical data for this approach are promising,[103], [104], [105] with the caveat that inhibition of specific integrins (ανβ3 and ανβ5 by cilengitide, an antiangiogenic compound) has been linked to undesired pro-inflammatory and profibrotic effects.^106^ The Rho/ROCK signalling axis is another potential target for mitigating the cellular response to mechanical stimuli, with several inhibitors available. However, few studies have specifically investigated them in direct relation to mechanosensing. Statins can modulate this pathway by inhibiting post-translational modifications of Rho-GTPases, which are necessary for their activation and localization to the plasma membrane.^34^ Statins are known to improve endothelial dysfunction through a variety of mechanisms, among them Rho-GTPase-driven ones^107^ and their benefits in CLD have been abundantly demonstrated.[108], [109], [110] At the cellular level, simvastatin has been shown to improve matrix stiffness-induced endothelial dysfunction mediated by Rho activity.^111^ Atorvastatin has been shown to mediate senescence of activated HSCs *in vitro*,^112^ as well as to inhibit ROCK activity and increase eNOS levels and functionality *in vivo*.^113^^,^^114^ Downstream of Rho-GTPases, the ROCK inhibitors fasudil, ripasudil and, more recently, belumosudil, are approved in certain parts of the world for different diseases. In human CLD, the only trial investigating fasudil has demonstrated a significant acute haemodynamic response, likely due to its vasorelaxant properties.^115^ However, systemic effects on mean arterial pressure and systemic vascular resistance prevent its widespread implementation in this setting, pointing to the need to design more targeted inhibition strategies.^116^^,^^117^ At the transcriptional level, YAP/TAZ is one of the most investigated potential targets for modulating mechanoresponsiveness in cirrhosis and liver carcinogenesis.^30^^,^^96^ However, no clinical data is currently available.

## How to investigate the mechanobiology of portal hypertension

### Tissue stiffness

Tissue stiffness can be measured in several ways, both at the microscopic and the macroscopic level. In the clinical setting, tissue stiffness is measured for diagnostic and prognostic purposes by shear wave or magnetic resonance elastography. In the laboratory setting, the stiffness of tissues or different materials used for cell culture, as well as the stiffness of cells themselves, can be measured at macro- or microscopic scale by atomic force microscopy, pipette aspiration, shear rheometry, and several other methods.^118^ The stiffnesses of tissues in the human body have been established to range from very soft (1-3 kPa) for brain tissue,^118^ to very stiff (Gpa) for bone, with healthy liver having a mean stiffness of 2.3–4.6 kPa.^119^^,^^120^ In cirrhosis, liver stiffness increases significantly and changes in liver stiffness correlate both with progression and regression of the disease.

*In vitro*, several biophysical methods can be employed to mimic the conditions of healthy and diseased organs. One of the more common such methods uses hydrogels of different stiffnesses, which can be functionalized and coated with the desired ECM protein; cells can either be cultured on top of the hydrogel, in a 2D setting, or encapsulated within the hydrogel to mimic a 3D setting. The composition of such hydrogels is varied and the choice of material depends on each experimental question.^121^^,^^122^ Moreover, several techniques allow for temporal and spatial modulation of the polymerization degree of these hydrogels, thus enabling the study of cellular response to dynamic stiffening or softening of their substrate, as well as to different patterns or gradients.^31^^,^^89^^,^^123^^,^^124^ Generally, the stiffness of hydrogels used for *in vitro* research of liver cells is situated between 0.5-5 kPa for simulating healthy liver and 10-30 kPa for fibrotic/cirrhotic liver, with few exceptions where higher stiffnesses (60 kPa, corresponding to advanced, decompensated cirrhosis) have been used.

Moreover, force application can be made, for example, by microbeads attached to the cell (*e.g*., to an integrin), which can then be moved through magnetic or optical tweezers, thus generating a mechanical stimulus at the single-cell level. By using atomic force microscopy, isolated pressure can be applied to either the plasma membrane or the cell nucleus.^28^^,^[125], [126], [127], [128] To understand cellular responses to cell stretching, there exist systems which allow for the application of uniaxial or multiaxial, cyclic or static stretch to either single cells or cell sheets. However, liver endothelial cells are subjected to non-pulsatile flow, thus experimental models using cyclic stretch are not entirely translatable to what happens *in vivo* in CLD.

### Shear stress and hydrostatic pressure

Given the complexity of the sinusoids, in recent decades, the idea of creating a support able to mimic the sinusoidal milieu has gained attention. Recapitulating PH *in vitro* is challenging because together with stiffness, shear stress and hydrostatic pressure should also be applied in the co-culture system. Shear stress can be modulated by dynamic cell culturing, which enables the control of pressure, flow, and cell-cell communication. In 2014, our team developed the first device able to mimic the architecture and paracrine communication of the liver sinusoid as well as the shear stress in pathological conditions.^129^^,^^130^ These studies, together with those from other groups,^131^ demonstrated that microfluidics recapitulate key mechanical stimuli of the *in vivo* scenario.^114^ Liver-on-a-chip is a technology that can be scaled, connecting several organs together to understand organ cross-talk in liver disease.^132^^,^^133^ This is particularly important in PH, where extrahepatic manifestations like splanchnic hyperaemia or bacterial translocation, among the many components, contribute mechanically and biologically to disease progression.

### Modulation of mechanosensing/transduction

Furthermore, pharmacological strategies can be employed to modulate different known mechanosensing or mechanotransducing molecules. The most straightforward of these approaches is inhibition of cytoskeletal tension using molecules such as latrunculin B, cytochalasin D (actin polymerization inhibitors), and blebbistatin (myosin inhibitor), among others.^134^ For more specific research questions, Rho/ROCK inhibitors^135^ can be employed, as well as inhibition of initial stiffness sensing by integrin inhibitors or modulation of calcium flow through mechanosensitive ion channels. Mechano-effectors at the nuclear level can also be modulated, as exemplified by verteporfin, a YAP inhibitor.^135^ Moreover, genetic approaches can also be employed to knock-down or knock-in different molecules involved in mechanosensing pathways.

### Experimental challenges

Several issues arise when investigating mechanobiology in the liver. Firstly, there is no standardized range for the translation of the mechanical inputs that the liver receives during development of CLD into *in vitro* models. When liver stiffness is measured in the clinical setting, the obtained value comprises the rigidity of the ECM and cells, as well as other factors, such as cholestasis, inflammation, and blood flow. How cells perceive these different inputs is challenging to dissect in an *in vitro* setting. This has resulted in the use of a wide range of substrate stiffnesses in cell experiments, starting from the 0.1-1 kPa measured in decellularized liver ECM,^68^ up to values of 10-30 kPa,^67^ as translated from the clinical setting. This makes between study comparisons difficult. Besides the stiffness itself, cell behaviour can be altered by modulating the biochemical properties of the scaffold. This makes decellularized scaffolds a suitable system when compared with artificial scaffolds. As an example, mesenchymal stem cells have been shown to respond differentially to soft *vs.* stiff PAA, whereas polydimethylsiloxane gels do not induce any differential response in the same conditions.^136^^,^^137^ However, PAA gels cannot mirror the native composition of the matrisome.

Secondly, the results obtained *in vitro* by applying mechanical stimuli are more difficult to translate to the *in vivo* setting, compared to the research of purely biochemical pathways, and they generally are merely correlated to observations made *in vivo*. The pharmacological or genetic manipulations described previously are hard to employ in an *in vivo* setting due to their wide-ranging effects.

Finally, attention must be given to the phenomenon of mechanical memory of cells when designing *in vitro* mechanobiology experiments. Although this concept is not fully understood yet, several studies have described an influence of past exposure to high stiffness on cell behaviour even after they are exposed to soft substrates.^25^^,^^89^^,^^90^ This raises the question of whether cell lines expanded and passaged on classic tissue culture polystyrene, which has an extremely high stiffness, will respond in the same way to a change in substrate rigidity as cells isolated freshly from a soft organ, such as the liver.

Another important issue is the cumulative effect of shear stress with stretching on specific pathways. NO as well as KLF2 can be modulated by stiffness and shear stress, which complicates the ability of *in vitro* models to recapitulate *in vivo* disease models. There is not yet a standardized system that can modulate shear stress and stiffness to mimic the progression of the disease. However, if we consider these mechanical stresses separately, there has been marked innovation in experimental models in the past few years. New *in vitro* platforms have provided opportunities to uncover the isolated contribution of each applied force, facilitating the understanding of the role of individual pathways and therefore of their modulation. These systems go from the basic 2D co-culture systems^114^^,^^138^ to the more advanced 3D cultures[139], [140], [141], [142], [143] depending on the type of stimulus that is required. However, there remain several limitations to 3D systems, including the control of sinusoidal regeneration and generation of blood vessels, and recapitulating nutrient availability and gas exchange. This was partially overcome by the application of large vessels as scaffolds for LSECs.^144^^,^^145^ A valuable alternative for the study of LSEC/HSC cross-talk in the presence of shear stress is the liver-on-a-chip system.^129^^,^^146^

## Conclusions

Mechanical stimuli and cellular mechanobiology balance tissue and organ physiology through a complex set of information and instructions, which cells continuously read and to which they respond. Chronic inflammation and injury in cirrhosis drive liver cell dysfunction, leading to excessive ECM deposition, sinusoidal pseudo-capillarization, vascular occlusion, and parenchymal extinction. This results in marked microarchitecture remodelling, altering environmental mechanical forces and triggering a progressive process of liver fibrosis, which becomes irreversible at a certain stage of the disease. PH is characterized by the alteration of several mechanical forces acting extra- and intrahepatically. Therapeutic approaches targeting the intrahepatic contribution to PH must rebalance the intracellular forces exerted by the cytoskeleton and promote the balanced remodelling of the liver microarchitecture. Reciprocal communication between cells and their niches represents a crucial target for novel therapeutic strategies against the progression of liver fibrosis.

## Financial support

S.S. is supported by the EASL Juan Rodés PhD Student fellowship, and the Swiss 10.13039/501100012451Liver Foundation. S.G.-M. is supported by the Sara Borrell program from Instituto de Salud Carlos III. A.B. is supported by the 10.13039/100000001Swiss National Science Foundation (320030_189252). J.G.-S. is supported by the 10.13039/501100004587Instituto de Salud Carlos III (FIS PI20/00220 and DTS22/00010, co-funded by the 10.13039/501100000780European Union), the 10.13039/501100015755CIBEREHD, the Swiss National Science Foundation (320030_189252), the 10.13039/501100004784Novartis Foundation for Medical-Biological Research, the Foundation Suisse Contre le Cancer du Foie and the AGAUR-Generalitat de Catalunya (2021 SGR 01322 & 2021 PROD 00036). CIBEREHD is funded by the Instituto de Salud Carlos III.

## Authors’ contributions

EF, SS and SG-M contributed to this paper with conception, literature review and writing. CC participated in literature review and writing. JB and AB made a substantial contribution to discussion of content and reviewed/edited the manuscript before submission. JG-S conceived, coordinated the writing, and critically reviewed and edited the manuscript.

## Conflict of interest

The authors declare no conflict of interest.

Please refer to the accompanying ICMJE disclosure forms for further details.
